# Assessment of bacteriocin production by clinical *Pseudomonas aeruginosa* isolates and their potential as therapeutic agents

**DOI:** 10.1186/s12934-024-02450-w

**Published:** 2024-06-13

**Authors:** Hamed Charkhian, Ehsan Soleimannezhadbari, Amin Bodaqlouei, Lida Lotfollahi, Hajie Lotfi, Nesa Yousefi, Ehsan Shojadel, Zafar Gholinejad

**Affiliations:** 1 Young Researchers Club, Urmia Branch, Islamic Azad University, Urmia, Iran; 2 Department of Pharmaceutical and Biomolecular Science, Faculty of Pharmaceutical Science, University of Milan, Milan, Italy; 3 Department of Microbiology and Virology, School of Medicine, Urmia University of Medical Sciences, Urmia, Iran; 4 Cellular and Molecular Research Center, Research Institute for Prevention of Non-Communicable Disease, Qazvin University of Medical Sciences, Qazvin, Iran; 5 Department of Pharmaceutical Biotechnology, Faculty of Pharmacy, Urmia University of Medical Sciences, Urmia, Iran; 6 Department of Medical Laboratory Science, Urmia Branch, Islamic Azad University, Urmia, Iran

**Keywords:** S-type pyocin, *Pseudomonas aeruginosa*, Bacteriocin, Antimicrobial pitied, qRT-PCR

## Abstract

**Introduction:**

Bacterial infections and the rising antimicrobial resistance pose a significant threat to public health. *Pseudomonas aeruginosa* produces bacteriocins like pyocins, especially S-type pyocins, which are promising for biological applications. This research focuses on clinical *P. aeruginosa* isolates to assess their bacteriocin production, inhibitory spectrum, chemical structure, antibacterial agents, and preservative potential.

**Methods:**

The identification of *P. aeruginosa* was conducted through both phenotypic and molecular approaches. The inhibitory spectrum and antibacterial potential of the isolates were assessed. The kinetics of antibacterial peptide production were investigated, and the activity of bacteriocin was quantified in arbitrary units (AU ml^−1^). Physico-chemical characterization of the antibacterial peptides was performed. Molecular weight estimation was carried out using SDS–PAGE. qRT-PCR analysis was employed to validate the expression of the selected candidate gene.

**Result:**

The antibacterial activity of *P. aeruginosa* was attributed to the secretion of bacteriocin compounds, which belong to the S-type pyocin family. The use of mitomycin C led to a significant 65.74% increase in pyocin production by these isolates. These S-type pyocins exhibited the ability to inhibit the growth of both Gram-negative (*P. mirabilis* and *P. vulgaris*) and Gram-positive (*S. aureus*, *S. epidermidis, E. hirae, S. pyogenes*, and *S. mutans*) bacteria. The molecular weight of S-type pyocin was 66 kDa, and its gene expression was confirmed through qRT-PCR.

**Conclusion:**

These findings suggest that S-type pyocin hold significant potential as therapeutic agents against pathogenic strains. The Physico-chemical resistance of S-type pyocin underscores its potential for broad applications in the pharmaceutical, hygiene, and food industries.

## Introduction

Infections caused by a number of bacteria and the escalating antimicrobial resistance pose a major threat to public health and human life [1–3]. Pathogenic bacteria often become resistant to various types of antibiotics due to their inherent characteristics, such as the limited permeability of their outer membrane, the presence of multiple drug efflux pumps, expression of antibiotic-degrading enzymes (e.g. Carbapenemases, β-lactamases), alteration of growth state (e.g. Biofilm formation), mutations and/or acquisition of resistance genes [4]. It is projected that due to the increasing trend in antibiotic resistance [5], the mortality rate of bacterial infections will surpass the total deaths from all types of cancer [6, 7]. This situation is expected to place a financial burden exceeding 100 trillion dollars on healthcare systems by 2050 [7]. Moreover, the widespread use of antibiotics in the livestock and poultry industry has made these animals susceptible to antibiotic-resistant bacterial strains [8, 9], thereby contributing to the proliferation of antibiotic resistance in environmental and animal pathogens. These pathogens can be transmitted to humans through the food chain, water, or contact with animals [9–11].

The production of over 23,000 secondary metabolites by diverse microorganisms has made them a focal point in the search for new antibacterial agents [12–16]. Secondary metabolites, typically synthesized from primary metabolites in response to specific conditions like stress, enable interactions with the environment [17–20]. Antibacterial secondary metabolites are categorized into two main groups by molecular weight: (I) low molecular weight (e.g., hydrogen peroxide, organic acids); and (II) high molecular weight (e.g., bacteriocins and/or bacteriocin-like inhibitory substances (BLIS)) [21–23]. Bacteriocins play a crucial role in defensive and competitive mechanisms. These extracellular toxins, produced by ribosomes, work by inhibiting or killing rival strains (those close to the main microorganism), ultimately enhancing survival by securing biological living space and improving access to nutrients [24–28].

*Pseudomonas aeruginosa (P. aeruginosa)* is recognized as a highly prevalent bacterial pathogen. This opportunistic Gram-negative gammaproteobacterium is capable of producing an array of extracellular products which ultimately serve to facilitate its spread [29]. Since 90% of *P. aeruginosa* strains are capable of producing at least one type of bacteriocins, they are an excellent model for protein based antibiotic investigation [28]. Bacteriocins produced by *P. aeruginosa* capable of inhibiting other strains are called pyocins, which are classified into three groups: S, R, and F. S-pyocins are large polypeptides with multiple domains. They include a cognate immunity protein that deactivates the catalytic domain of the active pyocin [28–31]. S-pyocins are soluble and sensitive to heat and proteases, a trait that distinguishes them from R- and F-pyocins [29, 30]. R- and F-type pyocins, also known as tailocins, originated as defective prophages with ancestral connections to P2 and lambda phages [28–31]. R-type pyocins possess a contractile structure but lack flexibility, whereas F-type pyocins are flexible but lack contractility [32]. Recently, M- and G-pyocins have also been discovered as members of the bacteriocins produced by *P. aeruginosa* [31]. Among the advantageous qualities of these pyocins are their ability to arrest of food spoilage, deter pathogenic microorganisms, and exhibit anti-inflammatory and antioxidant effects [33].

S-type pyocins, which have a size ranging from 30–40 to 90–100 kDa, belong to a group of molecules capable of efficiently crossing the impermeable outer membrane of various bacteria for the purpose of delivering cytotoxins which cause them death by different mechanisms [4, 34]. Those peptides can hold great promise for a range of biological applications. This potential is valuable to the pharmaceutical, healthcare, and food industries, particularly in addressing Multidrug Resistant (MDR) and Extensively Drug Resistant (XDR) infections [9, 23, 26, 35–37]. Therefore, it is crucial to isolate and identify strains that produce bacteriocins and/or BLISs, purify them, and conduct a comprehensive study aimed at enhancing resistance to infections and improving food hygiene. This research aimed to assess the capacity of clinical isolates of *P. aeruginosa* to produce bacteriocin compounds and the spectrum of inhibition these peptides demonstrate against pathogenic bacteria. Additionally, we explored their chemical structure and their potential utility as antibacterial agents and natural preservatives.

## Materials and methods

### Bacterial strains, media, and growth conditions

One hundred fifty *P. aeruginosa strains* were collected from various clinical sources in multiple hospitals in Urmia, Iran, between March and December 2021. These bacteria were preserved at − 20 °C with a 20% glycerol solution (w/v) in the microbiology laboratory of Urmia Medical School. To assess antimicrobial activity, we used indicator strains provided by the Pasteur Institute of Iran and the Persian Type Culture Collection (PTCC) in Tehran, Iran (Table 1). All strains were cultured in Mueller Hinton (MH) agar and incubated at 35–37 °C with shaking at 150 rpm when necessary.

**Table 1:** Indicator strains

No.	Standard strains	ATCC	MCI NO	Sources
1	*Staphylococcus epidermidis*	12228	1079	Pasteur Institute of Iran
2	*Escherichia coli*	25922	1101	
3	*Pseudomonas aeruginosa*	27853	1060	
4	*Proteus vulgaris*	6380	1063	
5	*Staphylococcus aureus*	29213	1075	
6	*Proteus mirabilis*	25933	1056	
7	*Shigella flexneri*	12022	1072	
8	*Klebsiella pneumoniae*	9997	1103	
9	*Burkholderia cepacia*	25416	1018	
10	*Streptococcus mutans*	35668	1091	
11	*Streptococcus pyogenes*	19615	PTCC: 1762	Persian Type Culture Collection
12	*Enterococcus hirae (Streptococcus faecium)*	9790	PTCC: 1238	

### Phenotypic identification of *P. aeruginosa*

The bacterial strains were identified through a series of tests following Bergey's Manual as a reference. These tests included morphological, physiological, biochemical, and carbon source utilization examinations. The isolates were subjected to biochemical analysis to determine oxidase and catalase activities, V-P and MR-VP tests, starch and gelatin hydrolysis, indole production, and citrate utilization. Additionally, morphological characteristics were assessed through Gram's staining and motility tests. Physiological characterization involved studying bacterial growth at 42 ºC and observing pigment formation [26, 37].

### Molecular identification of *P. aeruginosa*

#### DNA extraction

DNA extraction was performed using the boiling method. First, a certain number of colonies were suspended in a sterile tube containing sterile distilled water. The suspension was then centrifuged at 5000 rpm, and the supernatant was discarded, this process was repeated twice. The OD_600_ of the bacterial suspension was adjusted to 1.0, and it was heated for 10-min in a boiling water bath. Subsequently, it was immediately cooled on ice and centrifuged at 12,000 rpm for 5-min. The resulting supernatant served as the template DNA for the polymerase chain reaction (PCR).

#### PCR amplification of the 16 s rRNA gene

PCR was used to amplify the 16 s rRNA gene. A PCR mixture (25µL comprising of 12.5µL master mix, 0.5µL each of forward and reverse primers (20 pmol), 2µL template DNA (50 ng), and 9.5µL of nuclease-free water) was set up to amplify the 16 s rRNA gene using the primers (Table 2). Amplification was performed using Bio Intellectica, Canada. The PCR was programmed as follows: an initial denaturation at 95 °C for 5-min; 35 cycles of denaturation at 95 °C for 1-min; annealing at 58 °C and 72 °C, respectively, for 45-s each; and an extension at 72 °C for 60-s; followed by a final extension at 72 °C for 5-min. PCR products were separated by electrophoresis on a 1% agarose gel and detected by comparison with a 100 bp DNA ladder as a size marker under UV gel doc apparatus [38–40].

**Table 2:** Primer sequnces in PCR

No.	Primer	Sequence	Location	Annealing temp (°C)	Product size (bp)	Reference
1	PA-SS-F	GGGGGATCTTCGGACCTCA	189–206	58	956	[38–40]
2	PA-SS-R	TCCTTAGAGTGCCCACCCG	1124–1144			

### Initial screening of isolates with antibacterial peptides producing potential

To identify isolates that produce antibacterial Peptides, three colonies from each isolate were inoculated into 30 ml of MH Broth. These cultures were then incubated at 37 °C and 150 rpm for 24-h. After incubation, the resulting bacterial suspension was centrifuged at 4000 rpm for 5-min and filtered through a 0.45 µm biological filter. Subsequently, using the Spot Test method, 10 µl drops of supernatants were applied to plates that had been inoculated with indicator strains. The plates were examined for the presence of inhibition zones on the following day.

### Omission of microorganisms and causes of false positive results

The need for a significant volume of biological filters posed a major challenge to the progress of this study. To overcome this challenge, samples were subjected to high-speed centrifugation (10,000 rpm) three times, each for 5-min. After each centrifugation step, the resulting supernatant was transferred to a new sterile container. Due to the potential acidity of the supernatant, which could disturb the growth of indicator strains, Potassium hydroxide (KOH) (1 M) was added to neutralize this acidity. The catalase enzyme (2 mg/ml) was surcharged to eliminate any possible hydrogen peroxide. In addition, to denature extracellular proteases, the samples were incubated at 80 °C for 5-min. This resulting solution is referred to as a cell-free supernatant (CFS).

### Antibacterial potential and inhibitory spectrum in the absence of an inducing agent

The Agar Well Diffusion (AWD) method was employed for this purpose. Microbial suspension with a turbidity of 0.5 McFarland was prepared from indicator strains and inoculated on the agar plates by the lawn culture method. Then, wells (6 mm) were made at appropriate intervals. 50 µl of CFS was added to each well. To ensure uniformity in CFU/ml among all isolates, the OD600 of each sample was adjusted to 0.1 when starting the culture to achieve CFS. The plates were then incubated at 37 °C for 24-h. The diameter of the clear zone surrounding each well indicated the extent of antimicrobial activity [23].

### Antibacterial potential of isolates in the presence of an inducing agent (mitomycin C)

The method is akin to the previous procedure [2–5], with the distinction that at hour 20, we introduced mitomycin-C (Sigma-Aldrich) at a final concentration of 1 µg/ml to the bacterial cultures. These cultures were then incubated for an additional 4-h.

The three indicator strains that exhibited the highest sensitivity to CFSs, as well as the three bacterial isolates showing the greatest antibacterial potential, were selected for the subsequent phases of the study.

### Kinetics of antibacterial peptides production

To monitor bacteriocin production throughout the growth cycle, selected isolate suspensions were incubated with an initial OD_600_ of 0.1 at 37 °C and 150 rpm. Bacterial growth was assessed by measuring OD_600_ at 2-h intervals. Simultaneously, the antibacterial activity of CFSs from the isolates against indicator strains (*S. epidermis*) was evaluated using the AWD method at the same time points. This allowed us to pinpoint the onset and progression of antibacterial metabolite production over time [28].

### The activity of bacteriocin in arbitrary units (AU ml^−1^)

Due to the lack of standard reference compounds for bacteriocins, their antibacterial activities using Arbitrary Units (AU) are commonly employed to express. This approach is semi-quantitative. The AU is calculated as the reciprocal of the most diluted bacteriocin concentration that continues to result in a clear inhibition zone on the agar medium inoculated with indicator strains. AU of bacteriocins was determined without purification and detected by Spot Test. To achieve this, twofold serial dilutions (1/2, …, 1/248) were prepared from CFSs in MH Broth (pH = 7.0). 10 µl drops of each dilution were applied to plates that had been inoculated with indicator strains (*S. epidermidis, P. mirabilis, P. vulgaris* and *P. aeruginosa*). The plates were incubated at 37 °C for 24-h. The antibacterial activity was shown as AU ml^−1^ and calculated using the following formula [23, 41, 42]:

$${\text{Arbitrary \, Units}}({\text{AU}}/{\text{ml}}), = \,( {\text{Reciprocal \, of \, the \, last \, Inhibitory \, Dilution}}/{\text{Volume \, of \, CFSs}}( {\text{ml}}))\, \times \,{1}0^{{{-}{2}}}$$

### Physico-chemical characterization of antibacterial peptides

#### Impact of enzymes on antibacterial peptides activity

The sensitivity of the antibacterial compounds present within CFSs to enzymes was evaluated by treating for 2-h with Proteinase K (to assess the presence of protein), Lipase (to assess the existence of triglycerides) and α-amylase (to assess the presence of carbohydrates) with final concentrations of 1 mg/mL, at 37 °C and a pH of 7.0. Enzymes were deactivated by incubating the samples at 80 °C for 5-min [43]. Changes in the inhibition zone diameter in AWD method were compared to the control (without enzymes) to assess sensitivity.

#### Impact of heat on antibacterial peptides activity

To determine the effect of heat on the antibacterial peptides, samples were incubated at 50, 70 and 100 °C for a duration of 2-h. AWD method was used to determine changes in the antibacterial efficacy of the samples.

#### Impact of pH on antibacterial peptides activity

The impact of pH on the efficacy of the antibacterial peptides was assessed by adjusting the pH of the samples using 1 M KOH and Hydrochloric acid (HCL) to achieve pH levels of 2, 4, 6, 8, 10, and 12. After incubating at room temperature for 2-h, the samples were returned to a neutral pH. The AWD method was employed to assess changes in the antibacterial effectiveness of the samples [23, 44].

#### Impact of organic solvents on antibacterial peptides activity

Organic solvents have carbon-based molecular structures capable of dissolving or dispersing one or more other substances to create a solution. To investigate the impact of organic solvents on the antibacterial peptides, samples were treated with Acetone, Ethanol, Methanol, Ether, Xylene, Chloroform, and Toluene-80 with a final concentration of 5%. After incubating at room temperature for 2-h, the AWD method was employed to evaluate the changes in antibacterial efficacy [45].

#### Molecular weight estimation using SDS–polyacrylamide gel electrophoresis (SDS–PAGE) and related antibacterial assay

To facilitate the work method, instead of purifying using ammonium sulfate and doing dialysis, the suspensions of 24-h culture of selected isolates (A18, B94 and C130) were innovatively lyophilized. 0.2 g of the lyophilized material was reconstituted in 1 mL of TRIS/HCL buffer (10 mM; pH = 8), and lysozyme (final concentration 2.5 mg/ml). The suspension was agitated at 60-75 rpm for 30-min at room temperature. After another 30-min of shaking, the concentration of protein was measured by Bradford Assay Kit (Navandsalamat, Iran) [46, 47]. SDS–PAGE was conducted using the standard protein marker (range 11 kDa to 245 kDa/Sinaclon, Iran). The running gels contained 20% m/v acrylamide, and the stacking gels had 5% m/v acrylamide. After finishing the electrophoresis, the gel was cut into two parts. One-half of the gel was stained with Coomassie Brilliant Blue [48].

The other half of the gel was treated with a solution of 20% isopropanol (v/v) and 10% acetic acid (v/v) for 2-h followed by washing with distilled water for 4-h. The gel was placed on MH agar and overlaid with MH with agar (0.6%) containing indicator strains (*S. epidermis*). After 24-h incubation at 37 °C, the presence of an inhibition zone was checked [49].

### Gene expression

#### Total RNA extraction and cDNA synthesis

Total RNA was extracted using the phenol–chloroform method. To extract RNA, 2 ml of the 24-h bacterial culture (A18, B94 and C130) was centrifuged at 4000 rpm for 5-min. The pellet was then washed with 2 ml of normal saline. Next, 1 ml of TRIzol reagent (Sigma-Aldrich) was added and incubated at 4 °C for 10-min. After adding 200 µl of chloroform (Sigma-Aldrich), the sample was incubated again at 4 °C for 5-min. The mixture was centrifuged at 10,000 rpm for 5-min, separating it into three phases. To the uppermost phase, 500 ml of cold isopropanol (Sigma-Aldrich) was added. After vortexing for 1-min, the sample was centrifuged at 14,000 rpm for 5-min. The RNA pellet was then washed with cold 75% ethanol (Sigma-Aldrich). The pellet was dissolved in 20 µl of RNase-free water and stored at − 80 °C. Genomic contamination was eliminated using a DNase I Kit (Yekta Tajhiz Azma, Iran) following the manufacturer's instructions. The integrity of the extracted RNA was assessed by 1.5% agarose-TAE gel electrophoresis. The sample purity was determined using a nanodrop (Thermofisher, USA). For cDNA synthesis, 150 ng of extracted RNA with an absorbance ratio of 260/280 > 1.8 was used with the Parstous kit (Iran), following the manufacturer's instructions [50, 51].

#### Primer design and quantitative real-time PCR (qRT-PCR)

The specific primers for Pyocin S were designed using Oligo7 software (Table 3). The 16S rRNA gene was considered an internal control gene. qRT-PCR was performed using Kit Parstous/Iran following the manufacturer’s instructions. The reaction mixture contained 50 ng cDNA, 10p mol of primers, and 10 μL master mix that was adjusted to 20 μL with DEPC-treated water. Reactions were performed on the MIC real time PCR cycler (qPCR Cycler) (Bio Molecular Systems (BMS) co., Queensland, Australia) with the following cycle parameters: one cycle initial denaturation of 95 °C for 10 min and 45 cycles of 95 °C for 20 s, 60 °C for 30 s, and 72 °C 30 s. Data was reported as fold change from three individual experiments.

**Table 3:** Primers sequnces in qRT-PCR

No.	Product lenght	Sequnces 5ʹ → 3ʹ	Primers
1	Pyocin S (NCBI Sequence ID: D12708.1)	Forward: AGCTGTAATGAGAGATGG	206 bp
		Reverse: CATTCGGAAATCTTGGAC	
2	16 s rRNA gene	Forward: TGGAGCATGTGGTTTAATTCGA	159 bp
		Reverse: TGCGGGACTTAACCCAACA	

### Statistical analysis

All experiments were conducted in triplicate, and the data were denoted as mean ± standard error. Statistical analysis was performed using SPSS-19 software. Analysis of variance (ANOVA) and Duncan’s multiple-comparison tests were used to compare all results. Differences between means were considered significant when the confidence interval was smaller than 5% (P ≤ 0.05) [23].

## Results

### Identification of clinical isolates

All of the bacterial isolates were Gram-negative, obligate aerobic, non-spore-forming, rod-shaped bacterium that did not ferment carbohydrates. They were oxidase- and catalase-positive, while negative for indole and H_2_S production. Furthermore, those colonies emitted a characteristic scent resembling soap or grapes and produced green to brown pigments. The molecular confirmation of *P. aeruginosa* isolates was based on the presence of a 956 bp band on agarose gel.

### Screening of antibacterial peptides producing isolates

**Fig. 1:**
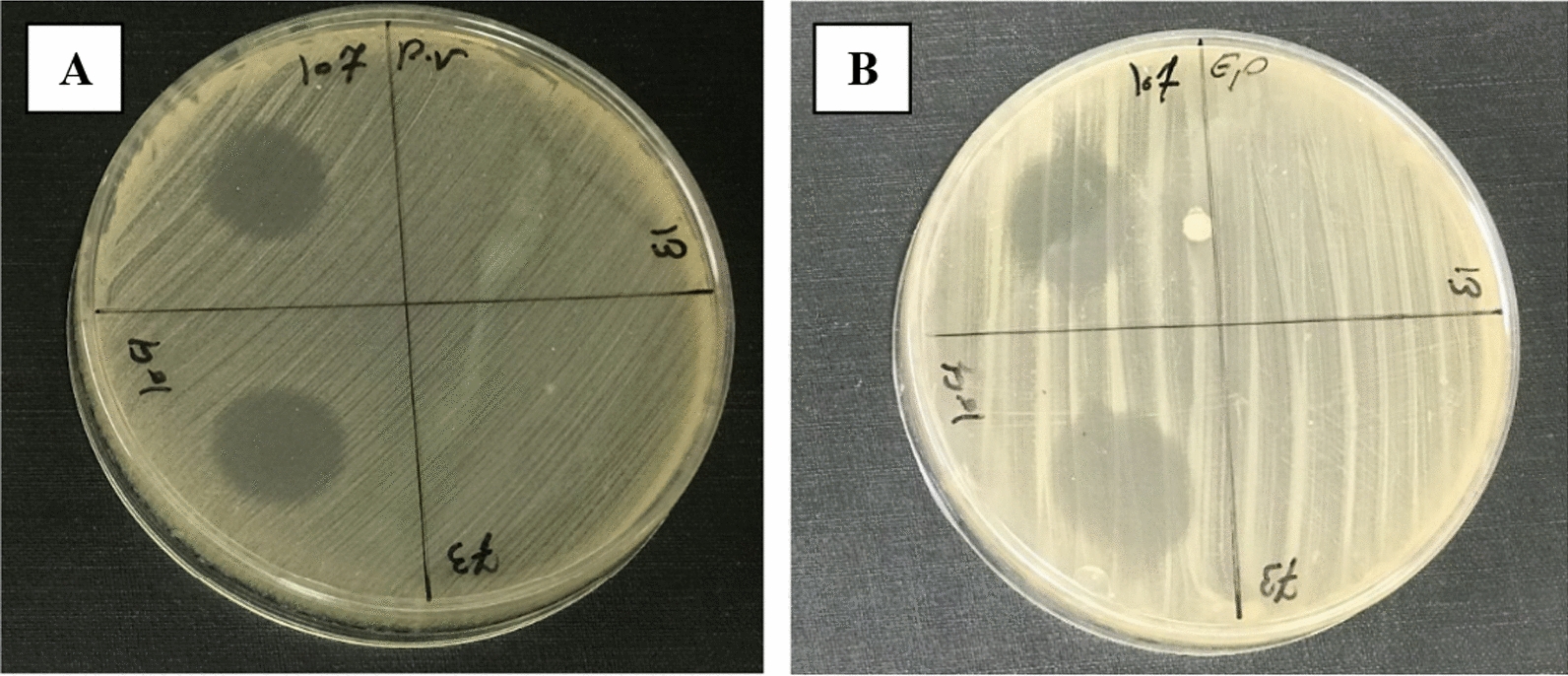
Spot test results for CFSs. **A**: *P. vulgaris*, **B**: *S. epidermidis*. Spot test was performed by spotting clinical isolate CFSs of *P. aeruginosa* onto a lawn of indicator strain. The presence of an inhibition zone after 24-h indicates the presence of antibacterial peptides in the CFSs. It is clearly observed that CFSs with codes C104 and C107 have created an inhibition zone

After eliminating factors leading to false positives, 13 out of the 150 *P. aeruginosa* isolates formed inhibition zones (Fig. 1; Table 4). It's worth noting that all 13 CFSs exhibited a similar inhibitory spectrum and inhibited the growth of *P. mirabilis*, *P. vulgaris* (Gram-negative), and *S. epidermidis*, *S. aureus*, *S. mutans*, *S. pyogenes*, and *E. hirae* (Gram-positive).

**Table 4:** Antibacterial potential of CFSs against indicator strains

Code	Indicator strains											
	S. ep	P. mi	P. vu	S. au	P. ae	E. co	K. pn	B. ce	S. en	S. mu	S. py	E. hi
A18	** + **	** + **	** + **	** + **	−	−	−	−	−	** + **	** + **	** + **
B79	** + **	** + **	** + **	** + **	−	−	−	−	−	** + **	** + **	** + **
B92	** + **	** + **	** + **	** + **	−	−	−	−	−	** + **	** + **	** + **
B94	** + **	** + **	** + **	** + **	−	−	−	−	−	** + **	** + **	** + **
B95	** + **	** + **	** + **	** + **	−	−	−	−	−	** + **	** + **	** + **
C104	** + **	** + **	** + **	** + **	−	−	−	−	−	** + **	** + **	** + **
C107	** + **	** + **	** + **	** + **	−	−	−	−	−	** + **	** + **	** + **
C108	** + **	** + **	** + **	** + **	−	−	−	−	−	** + **	** + **	** + **
C115	** + **	** + **	** + **	** + **	−	−	−	−	−	** + **	** + **	** + **
D121	** + **	** + **	** + **	** + **	−	−	−	−	−	** + **	** + **	** + **
D126	** + **	** + **	** + **	** + **	−	−	−	−	−	** + **	** + **	** + **
D130	** + **	** + **	** + **	** + **	−	−	−	−	−	** + **	** + **	** + **
D133	** + **	** + **	** + **	** + **	−	−	−	−	−	** + **	** + **	** + **

*Staphylococcus epidermidis: S. ep / Proteus mirabilis: P. mi / Proteus vulgaris: P. vu / Staphylococcus aureus: S. au / Pseudomonas aeruginosa: P. ae / Escherichia coli: E. co / Klebsiella pneumoniae: K. pn / Burkholderia cepacia: B. ce / Salmonella enterica: S. en / Streptococcus mutans: S. mu / Streptococcus pyogenes: S. py / Enterococcus hirae: E. hi*

( +) indicates the presence of an inhibition zone

( −) indicates the absence of an inhibition zone

### Comparison of the antibacterial effects of CFSs

**Fig. 2:**
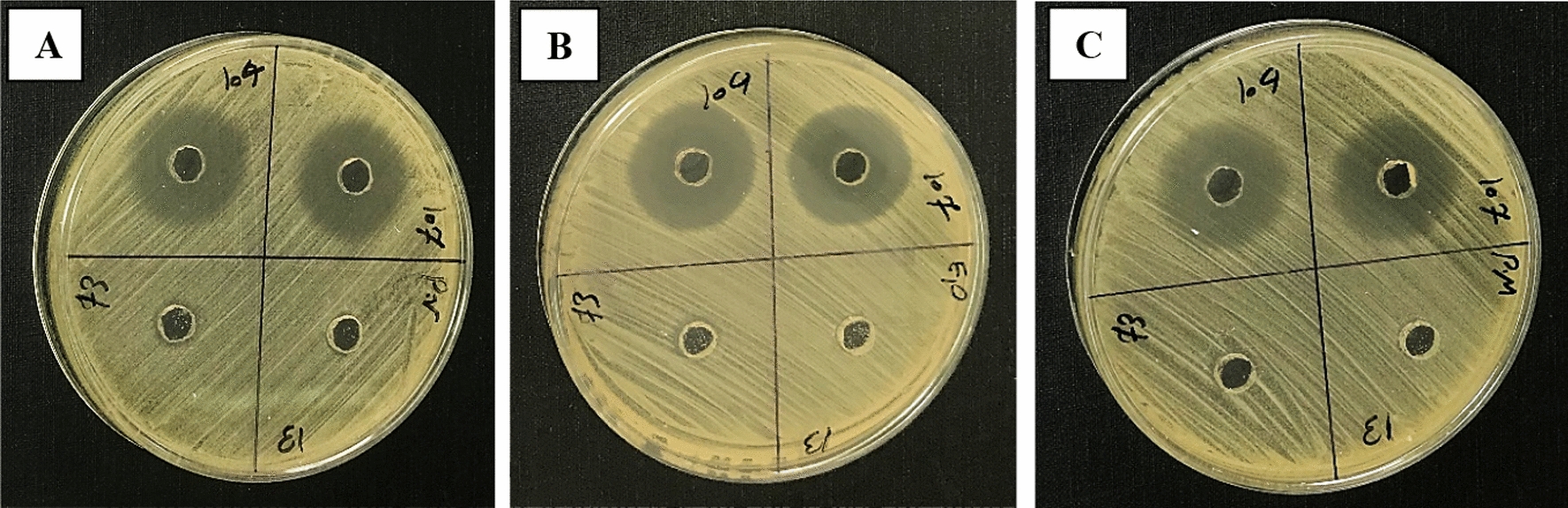
AWD method results for CFSs. **A** *P. vulgaris*, **B** *S. epidermidis*, **C** *P. mirabilis*. The presence of an inhibition zone after 24-h indicates the presence of antibacterial peptides in the CFSs. It is clearly observed that CFSs with codes C104 and C107 have created an inhibition zone

**Fig. 3:**
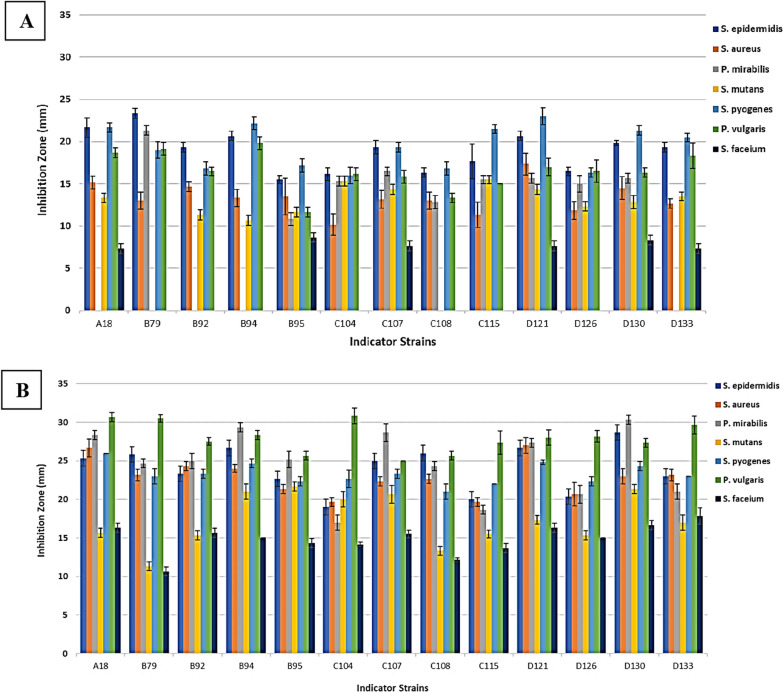
Antibacterial potential of CFSs in the absence of an inducing agent (**A**) and in the presence of Mitomycin C (**B**) by AWD method (mm)

**Fig. 4:**
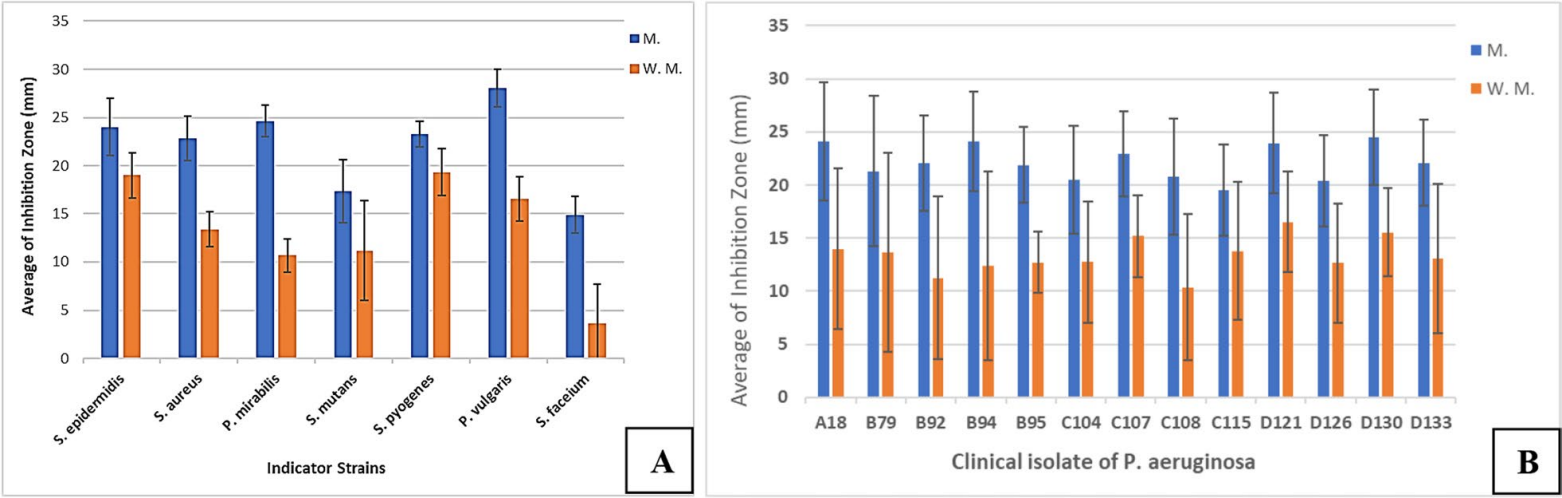
Comparison of microorganisms’ sensitivity to the antibacterial peptides produced by *P. aeruginosa* clinical isolates (**A**) and comparison of antibacterial potential CFS of Isolates (B). Figure 4A shows the average inhibition zone of each indicator strain in the vicinity of all peptides of *P. aeruginosa* clinical isolate (A18, B79, B92, B94, B95, C104, C107, C108, C115, D121, D126, D130, D133). Figure **B** shows the average total antibacterial effects of the peptides of each *P. aeruginosa* clinical isolates against all 7 indicator strains (*S. epidermidis, P. mirabilis, P. vulgaris, S. aureus, S. mutans, S. pyogenes* and *E. hirae*). In other words, the average inhibition zone of all 7 indicator strains in the vicinity of the antibacterial peptide of each isolate is reported. *W.m* without Mitomycin C, *M* Mitomycin C

**Fig. 5:**
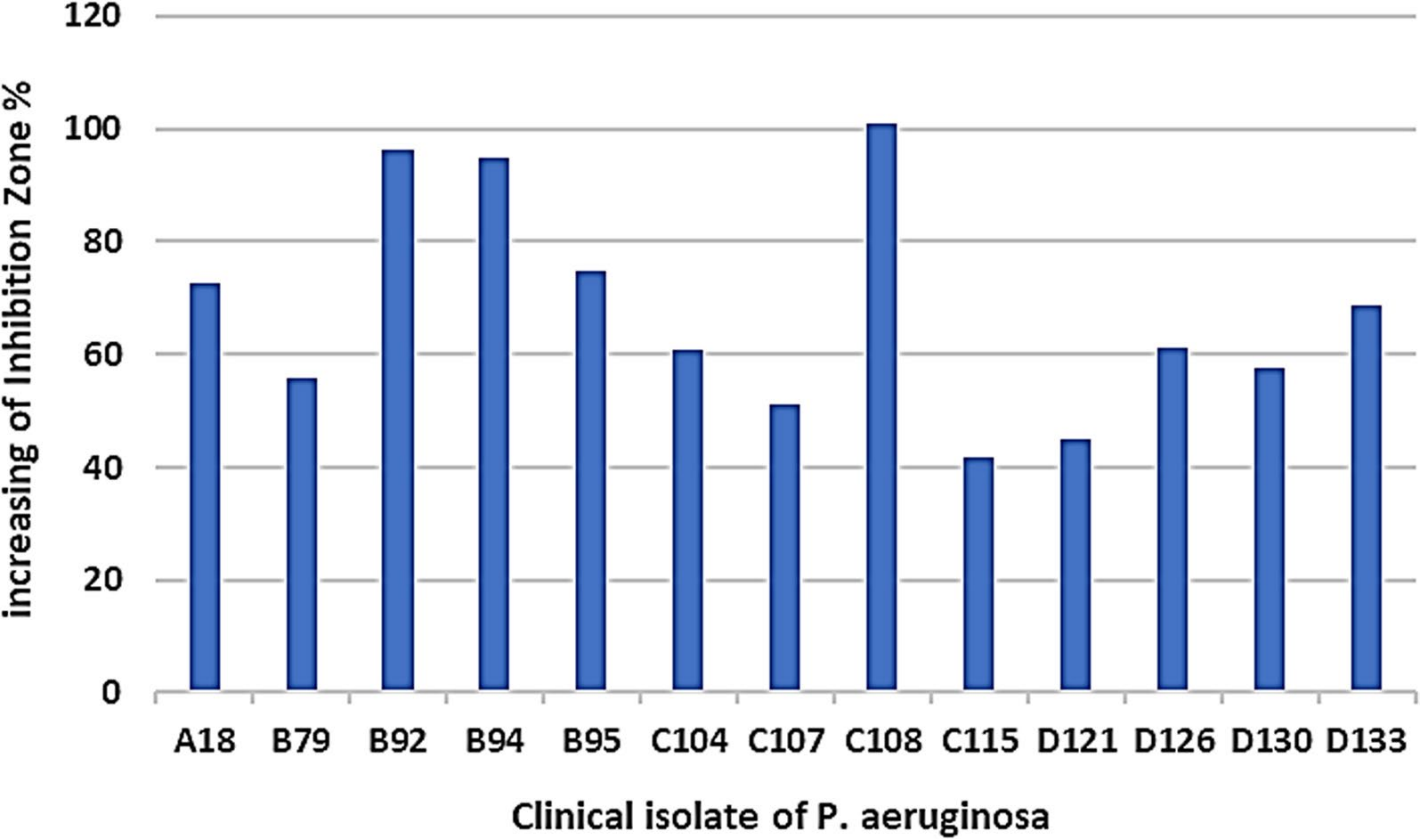
The increase in the percentage of inhibition zone after 4 h of treatment of the Clinical isolate of *P. aeruginosa* with Mitomycin C. Mitomycin C, as an inducer of pyocin production, significantly boosted antibacterial peptides production by an average of 67.78%. The most substantial increase was observed in isolate C108 (100.8%), while C115 showed the smallest increase (41.77%)

AWD method offered a more precise means of comparing the antibacterial potential of CFSs (Fig. 2). Investigating the impact of mitomycin C on the secretion level of antibacterial peptides by *P. aeruginosa* clinical isolates was one of the major points of the at-hand study. In the absence of an inducing agent, *S. pyogenes*, *S. epidermidis*, and *P. vulgaris* were the most sensitive microorganisms to the antibacterial peptides produced by *P. aeruginosa* clinical isolates, respectively. After treatment with mitomycin C, *P. vulgaris*, *P. mirabilis*, and *S. epidermidis* exhibited the highest sensitivity to these peptides (Figs. 3, 4A). Furthermore, in the absence of this inducing agent, two isolates D121 and C108 produced the strongest and weakest antibacterial peptides, respectively. In contrast, isolates D130 and C115 exhibited the most and least aantibacterial effect after treatment with mitomycin C (Figs. 3, 4B). Mitomycin C, as an inducer of pyocin production, significantly boosted antibacterial peptides production by an average of 67.78%. The most substantial increase was observed in isolate C108 (100.8%), while C115 showed the smallest increase (41.77%) (Fig. 5).

### Kinetics of antibacterial peptides production

**Fig. 6:**
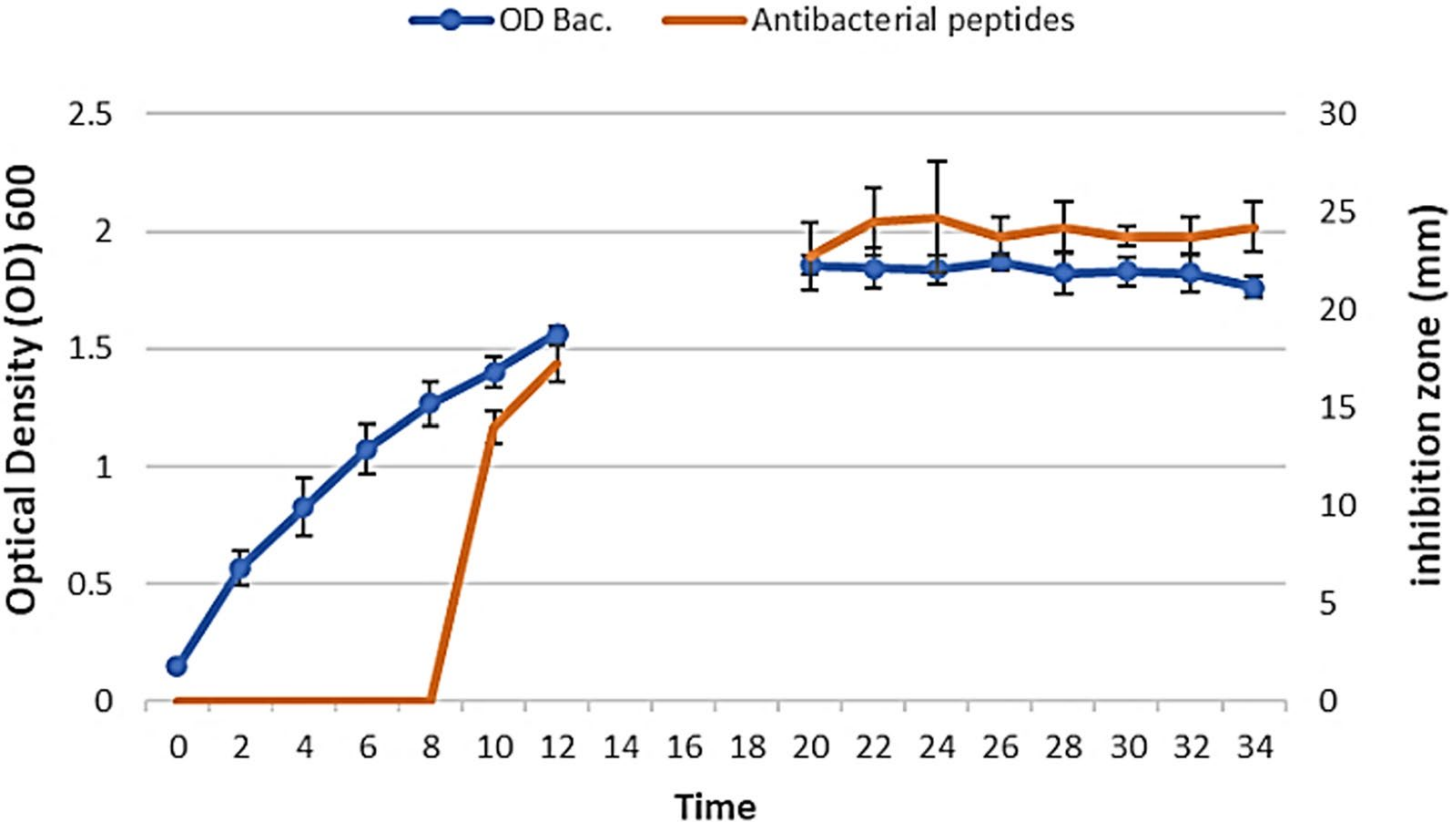
Bacterial growth curve and its relation with the level of antibacterial peptide release. The mean of three biological replicates with standard deviations is shown. The results are related to the isolate D121 against *S. pyogenes* in the absence of an inducing agent

In this test, the bacterial growth curve was plotted based on changes in turbidity caused by cell growth and division over time. As presented in Fig. 6, OD increases over time compared to the baseline (OD_0-h_). This increase indicates bacterial growth and division during the time. According to our observations, antibacterial peptides are not produced or are produced at undetectable levels during the initial 8-h (in the logarithmic and early exponential phase). Those peptide concentrations became detectable starting from the 10th-h (exponential phase) and their increase continued to rise until peaking at the 24th-h (stationary phase). It is notable to mention that at 6- and 8-h, a faint halo was observed around the wells that possibly indicated reduced bacterial growth. However, the size of these halos couldn't be accurately measured in millimeters.

### The activity of antibacterial peptides in AU

**Fig. 7:**
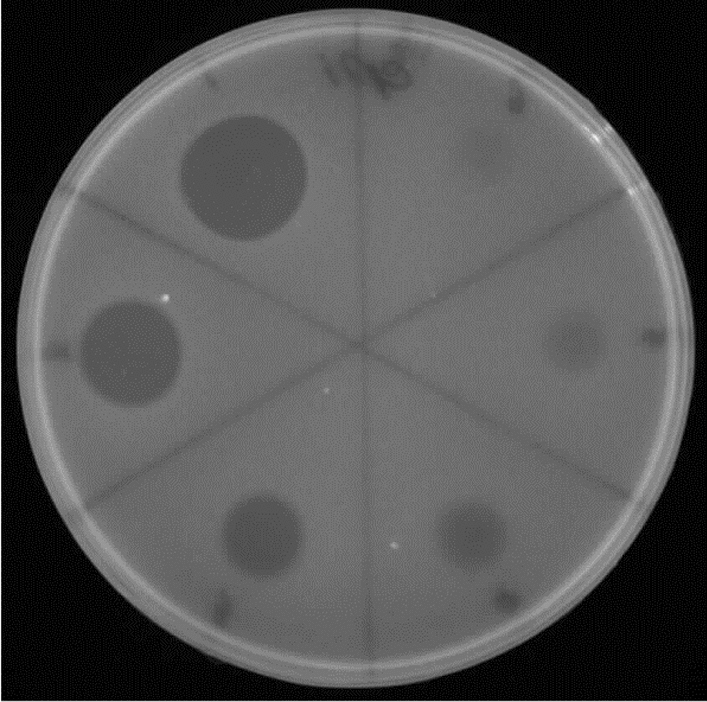
The sensitivity of *S. epidermidis* to the isolate D130's bacteriocins based on AU. The tested dilutions included 2^–1^, 2^–2^, 2^–3^, 2^–4^ and 2^–5^

In this test, the higher the number of AU, the stronger the antibacterial potency. The AU values were determined for three CFS samples against *S. epidermidis*, *P. vulgaris* and *P. mirabilis* (Table 5). The isolate D130's CFS was able to create an inhibition zone against *S. epidermidis* even at the sixth dilution, with an antibacterial agent concentration of 64 AU ml^−1^ at that dilution (Fig. 7).

**Table 5:** Comparison of the sensitivity of indicator strains to bacteriocins based on the Arbitrary Units (AU ml^−1^)

bacterial isolates	Indicator strains			
	S. ep	P. vu	P. mi	P. ae
A18	32	16	16	–
B94	32	16	16	–
D130	64	32	32	–

S. epidermidis: S. ep / P. mirabilis: P. mi / P. vulgaris: P. vu / P. aeruginosa: P. ae.

### Sensitivity of antibacterial peptides to heat, pH, enzymes and organic solvents

**Fig. 8:**
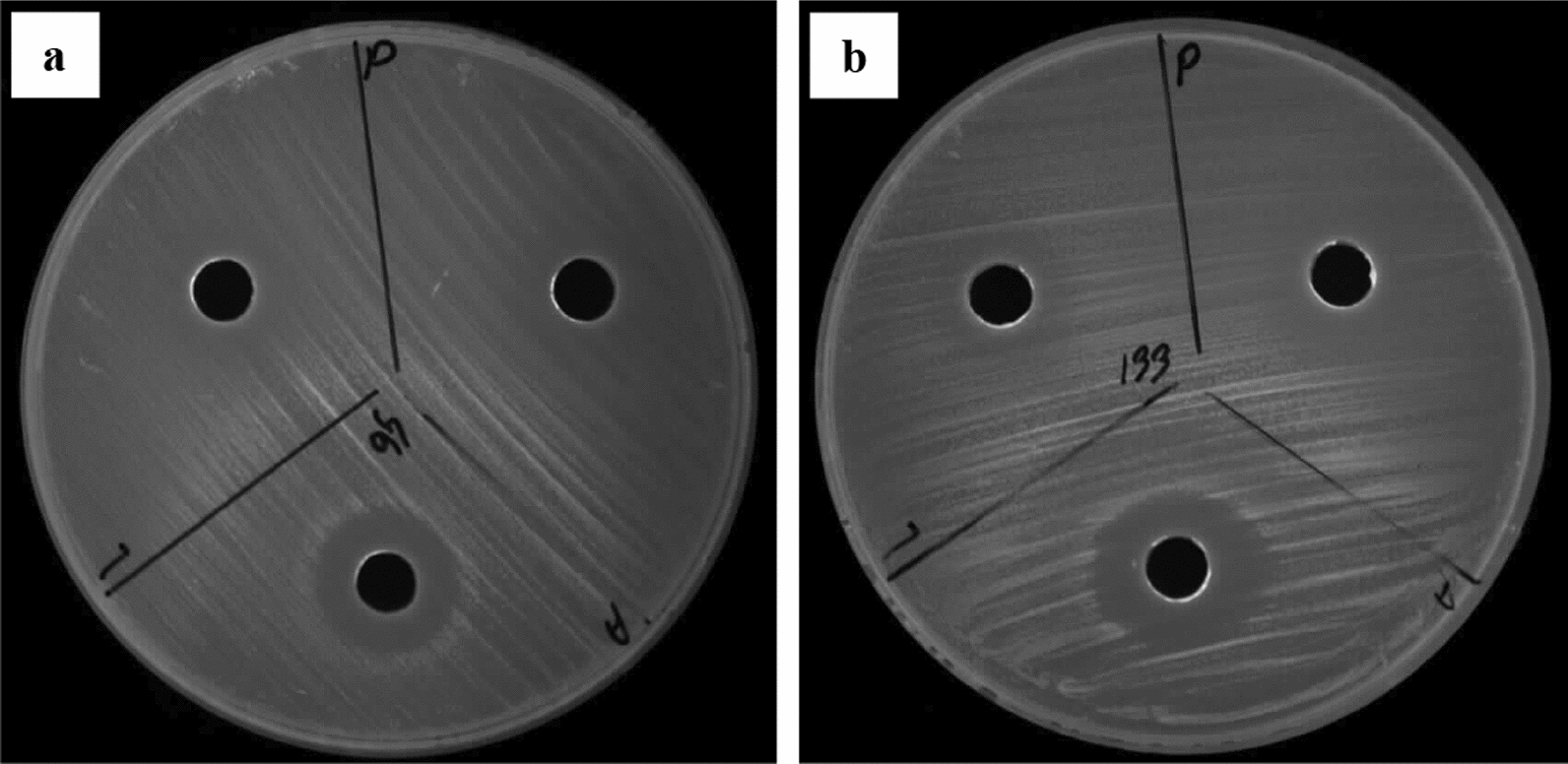
The effect of Proteinase K (to assess the presence of protein), Lipase (to assess the existence of triglycerides) and α-amylase (to assess the presence of carbohydrates) on the activity of antibacterial peptides. CFSs of the isolates treated with Proteinase K and Lipase enzymes could not cause an inhibition zone; This issue indicates the destruction of the protein and lipid structure of peptides. In this picture, the indicator strain is *P. vulgaris*, and the CFSs belong to isolates B95 (**a**) and D133 (**b**)

As summarized in Table 6, the antimicrobial properties of the CFSs did not change when treated with ethanol, acetone, methanol, ether, xylene, chloroform and toluene. Also, α-amylase enzyme was unable to inhibit the antibacterial potential of the samples, however, Proteinase K and Lipase enzymes were able to destroy this potential (Fig. 8). This further confirms the protein- and lipid-based structure of these antibacterial agents. In addition, the studied bacteriocins were found to remain stable when exposed to heat 50, 70 and 100 °C. The samples were also found to be resilient and retain their antibacterial properties in an extensive pH range, although extreme basic or acidic environments (pH: 2, 12) were found to limit their efficacy to some extent.

**Table 6:** The effects of different factors on the antimicrobial activity of bacteriocins

Treatment		Activity		
		C107	D121	D130
Enzymes (0.1 mg ml^−1^)	Proteinase K	–	–	–
	Lipase	–	–	–
	α-amylase	+	+	+
Organic solvents 10% (v/v)	Acetone	+	+	+
	Ethanol	+	+	+
	Methanol	+	+	+
	Ether	+	+	+
	Xylene	+	+	+
	Chloroform	+	+	+
	Toluene	+	+	+
Heat (15 min)	50 °C	+	+	+
	75 °C	+	+	+
	100 °C	+	+	+
pH	2	− (13.04% R)	− (13.39% R)	− (11.12% R)
	4	− (8.7% R)	+	− (93.33% R)
	6	+	+	+
	8	+	+	+
	10	− (8.7% R)	+	− (6.67% R)
	12	− (13.39% R)	− (13.39% R)	− (11.12% R)

CFSs of isolate C107, D121 and D130 were studied, and indicator strains included *P. vulgaris*. Untreated CFSs and the respective enzymes/chemicals were used as controls

(+): CFS is not affected by treatment and retains antibacterial activity;

(−): CFS is affected by treatment and loses antibacterial activity.

R: Reduction

### SDS-PAGE and molecular weight

**Fig. 9:**
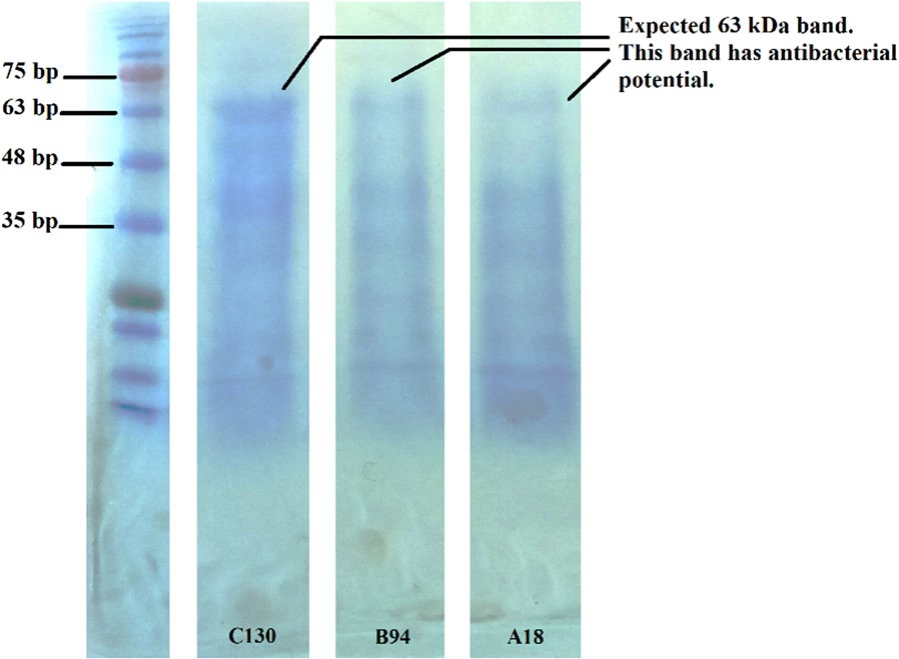
Molecular weight estimation of antibacterial peptides using SDS–PAGE

SDS-PAGE effectively characterizes proteins by their polypeptide molecular sizes. Gel electrophoresis was performed on the total protein samples, resulting in the appearance of numerous bands. Considering the resistance of F- and R-pyocins to proteases and the sensitivity of the antibacterial peptides identified in this study to proteinase K, previous research led us to anticipate bands with weights of 71–78 kDa [48, 52–54], 63-64 kDa [32, 54] and 56-58 kDa [4, 34, 47, 55] belonging to S-type pyocins. As depicted in Fig. 9, there is a band with a molecular weight of 66 kilodaltons visible in the gel. This band also exhibited antibacterial activity in the gel overlay assay (Figure not displayed).

### qRT-PCR

**Fig. 10:**
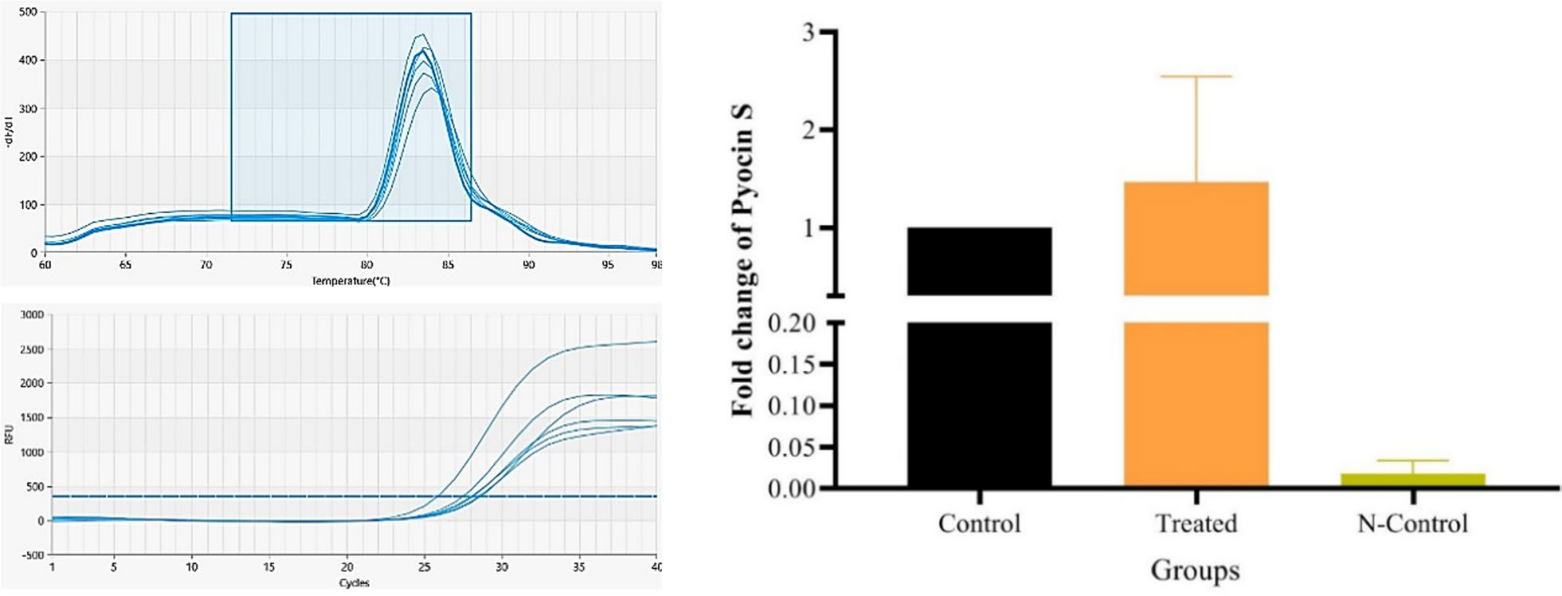
qRT-PCR analysis was conducted to confirm the expression of the Pyocin S gene. Control: 16 s rRNA gene, Treated: Pyocin S, N-Control: nuclease-free water

Based on the data obtained from the qRT-PCR procedure using the designed primers and their normalization with the universal gene, it can be deduced that 24-h after inoculating the microorganism, the gene responsible for S-pyocin production is being expressed at rates exceeding that of the universal gene (Fig. 10). This up regulation of S-pyocin gene was significant (P < 0.05).

## Discussion

The emergence and development of antibiotic resistance in pathogens has become a major threat to our everyday lives and the overall survival of our species. One of the most promising avenues for the development of novel antibacterial agents lies in the various microorganisms present across different biomes that, owing to their diverse genetic pool, are capable of producing undiscovered antimicrobial compounds. The identification and isolation of such agents are of paramount importance in our battle against pathogenic microorganisms.

In this study, clinical infection sources were utilized to isolate bacteria that produce antibacterial peptides. Previously, Shokri et al. [56], Iwalokun et al. [57], and Dingemans et al. [32] had successfully extracted antibacterial compounds from *P. aeruginosa* isolate. In another study, Naz et al. [49] identified pyocin SA189 in bacterial isolates collected from infected wounds. This attraction arises from the ability of *P. aeruginosa* strains to evolve in response to changes in environmental conditions. These evolutionary processes are due to genomic mutations and rearrangements that lead to phenotypic variations within populations [29]. Consequently, this adaptive capacity can enhance the potential for developing new antibacterial agents.

Based on the results obtained from the Spot Test and AWD method, and after ruling out potential causes of false positive observations, including extracellular proteases, pH, and hydrogen peroxide, it can be concluded that the current antibacterial agent is a secondary metabolite. Moreover, considering that antibiotics typically appear after the first day of cultivation, mainly in fungi, actinomycetes and spore-forming bacteria, and have broad-spectrum antibacterial activity [58–60], the production kinetics of the antibacterial peptides of *P. aeruginosa* isolates in this study (8-h after bacterial culture), which exhibit a narrow spectrum of lethality (7 bacterial families), strongly suggest that the antibacterial agent under investigation belongs to the bacteriocin class.

Regarding the production kinetics of antibacterial peptides by the selected isolates, antibacterial activity was detected in the CFS samples starting at 8-h- after culturing. This finding contrasted with the work of Doshi et al. [28], who reported the onset of pyocin secretion after 3-h of culturing. Similarly, Gholizadeh et al. [42] and Shokri et al. [56] also noted that the secretion of BLIS became detectable after 5- and 6-h, respectively. These incongruencies may be attributed to differences in the studied bacterial strains, culture medium, initial bacterial CFU/ml, shaking speed, and the structural characteristics and production rates of these substances. The common ground among all of the mentioned studies was that the concentration of the released peptides increased with time and peaked during the stationary phase after 24-h of incubation. Given that antibiotics are typically produced during the Stationary phase, while bacteriocins originate from ribosomal synthesis in the Log ph ase [61], the likelihood of the antibacterial peptides in this study being bacteriocins is higher. Microorganisms produce bacteriocins to obtain more resources in competition with others. Since there was no resource limitation in the early test hours, their production of bacteriocin might have been restricted.

Treatment with Mitomycin C increased the secretion of antibacterial peptides by an average of 67.78%. This data strongly supports the hypothesis that the antibacterial agent is a pyocin. Because pyocins are produced under stress conditions, and treatment with Mitomycin C (as DNA-damaging) can further activate their production [32, 62]. The SOS response, triggered in stress conditions, stimulates RecA's protease activity, leading to the deactivation of PrtR, the negative regulator of PrtN, which is the activator of pyocin gene expression [63–65].

The sensitivity of the peptides in CFSs to proteinase K and lipase suggests that these antibacterial agents have a protein and lipid structure. Moreover, since the clinical isolates were derived from *P. aeruginosa* strains, and S-type pyocins are known to be inherently sensitive to proteinases, it can be inferred that the antibacterial agents isolated in this study are indeed S-type pyocins. This conclusion was further validated through qRT-PCR analysis of the expression of the Pyocin S gene in *P. aeruginosa*. While there has been a recent discovery of several genes encoding S-type pyocins through the analysis of draft and whole-genome sequences of *P. aeruginosa* strains, it's worth noting that only six distinct S-type pyocins, inclusive of S1, S2, S3, AP41, S4, and S5, have been fully characterized and functionally examined to date [66–68]. The structure of S pyocins consists of three domains: a receptor-binding domain, typically located at the amino-terminal, a translocation domain, and a killing domain, positioned at the carboxy-terminal. S1, S2, S3 and AP41 pyocins exhibit DNase activity (similar to Colicin E2), S4 pyocins (similar to colicin E5) have tRNase activity, while S5 pyocins (similar to channel-forming colicins Ia and Ib) show pore-forming activity [32, 54, 62, 67, 69]. Pyocins use the proton motive force (PMF) to move across the cell envelope and deliver a cytotoxic domain [62]. Most S-type pyocins typically enter bacterial cells by binding to a TonB-dependent outer membrane receptor that plays a role in ferri-siderophore uptake. More precisely, pyocin S2 and S4 utilize the ferripyoverdine receptor FpvAI, pyocin S3 employs FpvAII, and pyocin S5 interacts with the ferripyochelin receptor FptA [32].

In this study, out of the 150 bacterial isolates, 13 isolates (8.7%) were observed to successfully inhibit the growth of seven indicator strains. Two of these indicator strains were Gram-negative (*P. mirabilis and P. vulgaris*), while the remaining five were Gram-positive (*E. hirae, S. pyogenes, S. mutans, S. aureus, and S. epidermidis*). In line with our findings, Doshi et al. [28] observed the sensitivity of Gram-positive bacteria such as *S. aureus* and the resistance of Gram-negative bacteria such as *E. coli* to the CFSs obtained from *P. aeruginosa*. Additionally, Iwolokum et al. [57] reported the sensitivity of bacteria such as *S. aureus, S. epidermidis*, and *Proteus* species to antibacterial metabolites produced by *P. aeruginosa*.

One of the notable observations was that the S-type pyocins did not inhibit the growth of indicator strain *p. aeruginsa* (ATTC 27853). Pyocins are synthesized as multi-domain proteins along with a highly specific immunity component. To be more precise, bacterial strains generating pyocins possess the respective immunity gene within the identical operon. This immunity component protects the producing strain from the antibacterial effects of the pyocin by binding to the C-terminus of the cytotoxic domain [27, 31, 62]. Hence, because these two bacteria share the same genus and species, their defense mechanisms against the cytotoxic domain of S-type pyocins are both active.

Unlike the observation made by Iwolokum et al. [57], the CFSs were incapable of inhibiting the growth of *K. pneumonia*. Furthermore, other Gram-negative indicator strains of this study including *S. enterica, E. coli,* and *B cepacia* were also immune from the cytotoxic effects of the S-type pyocins. The precise mechanisms by which pyocins enter bacterial cells are not completely clear. Nonetheless, what is clear is that pyocins precisely target particular receptors using their N-terminal sequences, and by interacting with these translocators within the outer membrane of bacteria, create energized import systems known as translocons. Furthermore, interacting with cell envelope components one after another, in other words, multiple protein–protein interactions are necessary for the import of pyocins. This specificity and complexity of enter mechanism of pyocins explain why they selectively kill specific strains of bacteria [4, 31, 70–73]. The appropriate translocation machinery acts as a two-sided blade in that although it may increase the chances of entering the cell in certain bacteria, it may render these agents ineffective against microorganisms lacking the necessary receptors. Furthermore, immunity protein genes can exist in isolation [31]. This implies that certain bacteria might have these genes independently, allowing them to develop immunity against pyocins.

These antibacterial peptides generally exhibit their bactericidal effects by penetrating the cell membrane, creating pores through the inhibition of peptidoglycan biosynthesis via lipid II degradation. Additional cytotoxic mechanisms might involve the suppression of enzymes, inhibition of nucleic acid and protein production, and the degradation of DNA or RNA. Various factors, such as amino acid sequence, polarity, charge, and shape, play a role in the adhesion of pyocins to the cell membrane and their subsequent entry [27, 74].

In another section of this study, the susceptibility of pyocins was examined to various physical and chemical factors. These factors are important to investigate because they can greatly influence the bioactivity of pyocins. Understanding their effects is crucial for potential applications in fields such as food preservation, probiotics, chemotherapy, and antibacterial agents [49]. The findings of this study indicate that pyocins displayed notable resistance to nearly all the physical and chemical factors we examined. Therefore, it can be concluded that pyocins possess inherent thermotolerance and remain stable across a range of pH levels, even in the presence of organic solvents.

The following studies are recommended to investigate the toxicity of pyocins in human cell lines. Upon being proven that bacteriocins do not pose a significant cytotoxic effect on human cells, they can be considered as a promising novel therapeutic and antibacterial agent against antibiotic-resistant microorganisms. Additionally, the resistance of bacteriocins to organic solvents, temperature fluctuations, and pH changes further enhances their potential for broad applications in the pharmaceutical, hygiene, and food industries.

One significant obstacle to the widespread use of pyocins is the potential emergence of pyocin-resistant mechanisms. To mitigate this issue, a possible solution is the creation of cocktails containing different pyocins that target diverse proteins. Therefore, the exploration of *P. aeruginosa* genomes for new pyocin genes is a crucial advancement in the development of this antibiotic class for therapeutic purposes (31).

### Conclusion

The current study's findings indicate that *P. aeruginosa* isolates obtained from clinical sources Can produce antibacterial agents. Their bactericidal activity is attributed to the secretion of bacteriocin compounds, which belong to the S-type pyocin family. The use of mitomycin C, an inducer of pyocin secretion, led to a significant 65.74% increase in pyocin production by these isolates. These S-type pyocins exhibited the ability to inhibit the growth of both Gram-negative (*P. mirabilis* and *P. vulgaris*) and Gram-positive (*S. aureus, S. epidermidis, E. hirae, S. pyogenes,* and *S. mutans*) bacteria. In this study, the molecular weight of pyocin S was determined to be 66 kDa, and its gene expression was confirmed through qRT-PCR. The findings indicate that S-type pyocin displayed notable resistance to nearly all the physical and chemical factors. These findings suggest that S-type pyocin holds significant potential as a therapeutic agent against infections and pathogenic strains, which can enhance treatment efficacy and reduce the financial burden on healthcare systems. Moreover, the resistance of S-type pyocin to organic solvents, temperature fluctuations, and pH fluctuations underscores its potential for broad applications in the pharmaceutical, hygiene, and food industries.

## Data Availability

The datasets produced and/or analyzed in this study can be obtained from the corresponding author upon reasonable request.
